# Ultrasound flow imaging for assessing cerebrovascular changes following focused-ultrasound blood-brain barrier opening

**DOI:** 10.7150/thno.98098

**Published:** 2025-09-22

**Authors:** Sua Bae, Stephen A. Lee, Seongyeon Kim, Fotios Tsitsos, Yangpei Liu, Elisa E. Konofagou

**Affiliations:** 1 Department of Biomedical Engineering, Columbia University, New York, NY, USA.; 2 Department of Radiology, Columbia University, New York, NY, USA.

**Keywords:** focused ultrasound, blood-brain barrier, ultrasound localization microscopy, ultrasound Doppler imaging, vascular monitoring

## Abstract

**Rationale**: Microbubble-mediated focused ultrasound (Mb-FUS) is a promising non-invasive technique for blood-brain barrier opening (BBBO), enhancing drug delivery and immunomodulation for brain disease treatments. In Mb-FUS, microbubble cavitation exerts mechanical stress on blood vessel walls. While cavitation is commonly used for monitoring, leveraging the vascular response to predict treatment outcomes remains unexplored. This study pioneers the use of ultrasound flow imaging with microbubbles to investigate the cerebrovascular changes induced by Mb-FUS and assesses the feasibility of this imaging technique for predicting BBBO treatment outcomes.

**Methods**: We utilized contrast-enhanced power Doppler (CEPD) and ultrasound localization microscopy (ULM) to monitor and quantify Mb-FUS-induced cerebrovascular changes in mice (n=4 without skull, n=12 with skull). The left hippocampus/thalamus regions were targeted for Mb-FUS BBBO. Pre- and post-FUS images were acquired, with continuous monitoring of CEPD intensity to ensure consistency in microbubble concentration. We observed changes in the number of microbubbles detected, their speeds, and vessel diameter after Mb-FUS.

**Results**: Reductions in blood volume, vessel diameter, and flow speed were observed in the sonicated regions. We demonstrated the transcranial capability of CEPD and ULM to detect Mb-FUS-induced vascular changes by observing linear relationships between the reductions in blood volume and flow, and the size of the opening or edema. Furthermore, local signal reduction detected by transcranial CEPD map spatially co-localized with the edema region identified in T2-weighted MRI.

**Conclusion**: We have developed a method to quantify changes in blood volume, flow speed, and vessel diameter following Mb-FUS using ultrasound flow imaging (CEPD and ULM) with microbubbles. For the first time, the blood vessels post-FUS were assessed by ultrasound flow imaging that visualizes associated vascular changes and potential damage. This technique not only holds potential for predicting treatment outcomes but also paves the way for a unified ultrasound-based system for both treatment and monitoring, with potential for future clinical translation.

## Introduction

Microbubble-mediated focused ultrasound (Mb-FUS) is a promising non-invasive treatment for the transient and localized blood-brain barrier opening (BBBO) to enhance drug delivery 1,2 and promote immune responses 3-5. The clinical translation of this treatment holds promise, as evidenced by recent successes of Mb-FUS for various diseases, such as Alzheimer's disease 6,7, Parkinson's disease 8,9, and brain tumors 10,11. The delivery of Mb-FUS treatment to the brain can be achieved using commercialized systems, including MR-guided FUS 6-9 and implantable FUS 11,12, as well as emerging systems such as neuronavigation-guided FUS 10,13 and ultrasound-guided FUS 14,15.

In this treatment, microbubbles are systemically administered, and focused ultrasound (FUS) induces rapid and nonlinear oscillations of microbubbles within a targeted volume of the brain. These oscillations, known as cavitation, exert mechanical forces to the blood vessel walls, causing the transient relaxation of tight junctions between endothelial cells and the increase of transcytosis and fenestration 16-18.

Not only does Mb-FUS increase BBB permeability, but it has also been shown to influence vascular dynamics. Optical microscopy through a cranial window revealed that Mb-FUS for BBBO induces transient vessel constriction and dilation in rodent brains 19,20. Cho *et al.* found that vasoconstriction is more prevalent than vasodilation in mice and the constrictions were typically maintained for 5-15 min. Burgess *et al.* showed that leakage of the dye through the vessel walls was accompanied by vasodilation, occasionally preceded by rapid vasospasm in Alzheimer transgenic mice 21.

In contrast to microscopy studies observing individual vascular morphology at a shallow depth (<0.3 mm), MRI studies captured the vascular response across the entire brain. Stupar *et al.* demonstrated a substantial reduction in cerebral blood flow in the sonicated hemisphere 30 min after FUS-induced BBBO in rats 22, accompanied by edema, using pseudo-continuous arterial spin labeling (pCASL). Additionally, a more recent study using pCASL MRI confirmed the transient reduction in blood flow following BBBO even in the absence of edema or hemorrhage 23. Furthermore, fMRI studies revealed that Mb-FUS can suppress the neurovascular response 24,25.

Despite various studies on vascular responses to Mb-FUS, to the best of our knowledge, ultrasound blood flow imaging has not yet been employed for monitoring or assessing FUS-induced BBBO. Ultrasound flow imaging offers significantly greater penetration depth compared to optical imaging and provides a more cost-effective option than MRI. In addition, this technique could be integrated into ultrasound-guided FUS systems, enhancing the portability and cost-effectiveness of BBBO treatments 14,26.

Ultrasound Doppler imaging has been utilized for transcranial blood flow imaging to study cerebrovascular structure and function 27. Additionally, microbubbles, also used for BBBO, can serve as a contrast agent to enhance imaging sensitivity through the skull 28. Ultrasound localization microscopy (ULM) with microbubbles can deliver high-resolution microvascular imaging below the ultrasound diffraction limit by localizing bubbles from hundreds of thousands of frames 29,30.

In this study, it is shown for the first time that contrast-enhanced power Doppler (CEPD) imaging and ULM can be utilized to transcranially monitor Mb-FUS-induced BBBO, using the same microbubbles concurrently with BBBO. We established a method to acquire CEPD and ULM for quantification of FUS-induced vascular changes in the presence of microbubbles, and estimated the changes in blood volume, vessel diameter, and flow speed via microbubble detection within the vessels. We conducted an open-skull study to ensure optimal image quality, and evaluated transcranial feasibility with intact skin and skull.

## Results

### BBBO through cranial window using Mb-FUS with PCI

**Figure 1:**
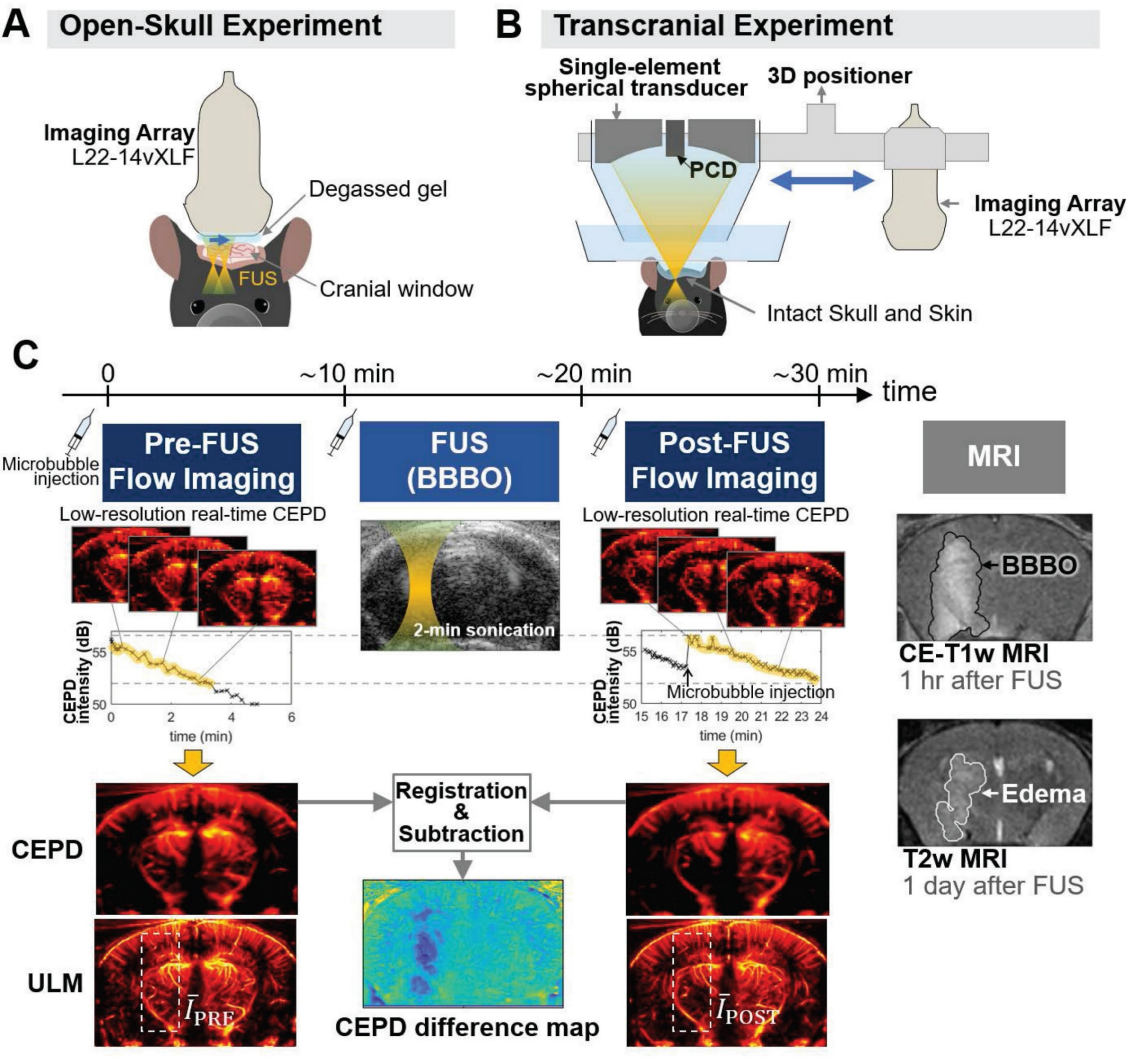
** Experimental setup and data acquisition for monitoring vascular changes following Mb-FUS**. (**A**) Open-skull experimental setup for optimal flow image quality, avoiding skull-induced acoustic attenuation and aberration. Theranostics ultrasound (ThUS) sequence was used to utilize a single imaging array transducer for both imaging and treatment. The sonication was monitored by power cavitation imaging (PCI). (**B**) Transcranial experiment setup for evaluating the transcranial feasibility. Traditional FUS sequence with a single-element spherical transducer and a passive cavitation detector (PCD) was used for BBBO and cavitation dose monitoring, and vascular images were obtained using the imaging array. (**C**) Acquisition of pre-FUS and post-FUS ultrasound flow images with similar microbubble concentrations and the contrast-enhanced T1-weighted (CE-T1w) and T2-weighted (T2w) MRIs. A 100-μL bolus of diluted microbubble solution was administered for both pre-FUS and post-FUS imaging sequences, as well as for FUS sonication. Monitoring of microbubble concentration in the mouse brain was achieved by real-time low-resolution contrast enhanced power Doppler (CEPD) images and their averaged intensity (i.e., CEPD intensity) over time. High-resolution CEPD and ULM images were reconstructed offline from the datasets with a similar range of CEPD intensity (yellow highlights in the CEPD intensity graphs) between pre- and post-FUS. CE T1-w MRI and T2w MRI scans were performed to identify BBBO and edema, respectively, which were then compared with ultrasound images.

In the open-skull study, the same linear array transducer was used for both FUS and imaging to ensure optimal imaging quality and precise alignment between the sonicated region and the imaging plane, as shown in Figure 1A. FUS was applied through a cranial window for 2 min with acoustic cavitation monitoring. Note that we applied five foci spanning a lateral distance of 0.5 mm to ensure sufficient coverage of the target region. The FUS pulse sequence used in the study is presented in Figure S1. The -6 dB region extended into both the cortical and thalamic areas, while the -12 dB region covered the entire depth of the brain (Figure S2).

**Figure 2:**
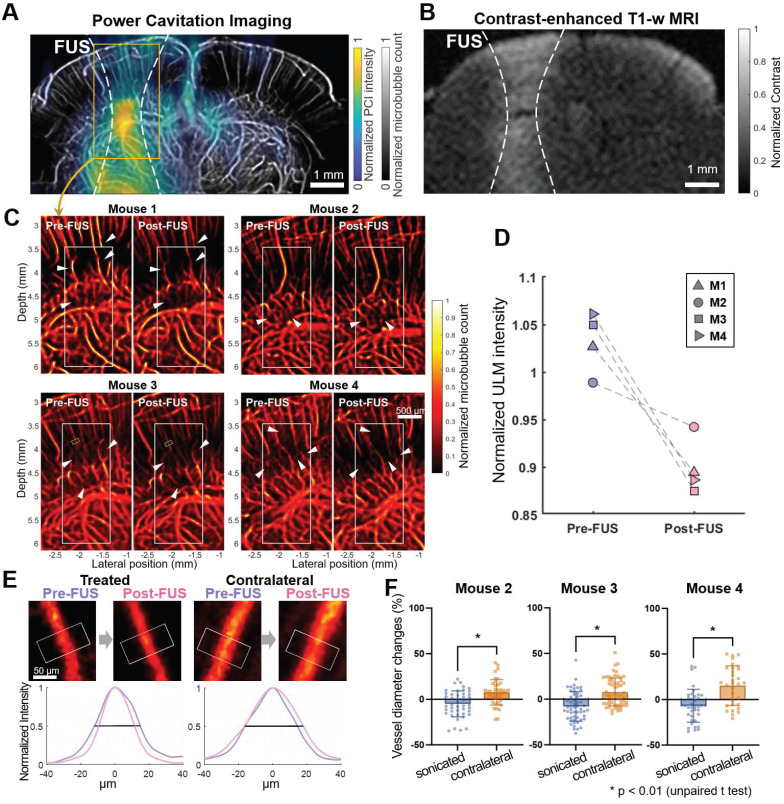
** Cerebrovascular changes after FUS in the open-skull experiments. A**) Cumulative power cavitation imaging (PCI) map obtained during FUS sonication overlaid on the vessel map (gray). **B**) Resultant BBB opening verified in contrast-enhanced T1-weighted MRI. In A and B, the -12 dB contour of the synthesized pressure field of 5 foci is indicated by white dashed lines. **C**) ULM intensity maps before and after FUS at the sonicated region. White boxes at the focus show the ROIs used for the mean intensity analysis. **D**) Mean intensity within the ROI (white box in C) normalized by the contralateral region. Normalized intensity decreased following FUS in all mice. **E**) Representative vessel in the sonicated and contralateral regions for diameter measurements before and after FUS. Fifteen cross-sections were obtained within the segment (white boxes in E and green boxes in C) and averaged to obtain a mean intensity profile. Its FWHM was measured as the diameter of the vessel. The full-width half-maximums of the mean intensity profiles of the pre-FUS (pink) and post-FUS (purple) were used for measuring the vessel diameter change. **F**) Vessel diameter changes after Mb-FUS in each mouse. Each data point represents the measurement from each vessel segment (* *p* < 0.01, unpaired t-test).

As shown in Figure 1C, ultrasound flow images were acquired approximately 10 min before and after Mb-FUS with similar microbubble concentrations. Figure 2A displays the cumulative cavitation energy map during the sonication, obtained by power cavitation imaging (PCI), overlaid on the vascular image acquired using ULM. A real-time PCI movie is available as supplementary video (Movie S1). The intensity of the PCI map corresponds to the number of acoustic cavitation events and their emission strength 31. The PCI map and video showed higher acoustic energy at the focus in the left hemisphere at (*x, z*) = (-2 mm, 5 mm). Overall, higher intensity was observed in denser vascular regions with larger vessels. BBBO was confirmed for all mice by the contrast enhancement observed in contrast-enhanced T1-weighted (CE-T1w) MRI (Figure 2B).

### Microbubble count reduction and vessel diameter change following Mb-FUS in the open-skull study

Figure 2C presents the ULM images of the sonicated brain region from four mice. The intensity (i.e., the number of detected microbubbles) of each ULM image was normalized by the mean intensity of the contralateral region. These images show a decrease in the microbubble count after FUS at the sonicated site, indicated by white arrow heads. The reduction in the microbubble signal was particularly pronounced in small arterioles/venules and capillaries in the dorsal hippocampus. The normalized intensity (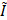) within the region-of-interest (ROI) centered at the FUS focus (white boxes in Figure 2C) decreased after Mb-FUS in all mice with an average percent change of -12.7% and a standard deviation of 4.5% (Figure 2D).

From the pre- and post-FUS ULM images, vessel segments were selected in both sonicated and contralateral regions from three mice, and the average vessel diameter was measured for each segment (Figure 2E). One mouse was excluded due to an insufficient number of datasets with matched CEPD intensity. The diameter of selected vessels ranged from 10 μm to 100 μm and their distributions in the sonicated and contralateral regions are presented in Figures S3A and S3B. While both vasoconstriction and vasodilation were observed in both hemispheres, a significant difference (*p* < 0.01) in vessel diameter changes was found in three mice between the treated and contralateral regions, as shown in Figure 2F (*t*-values = 4.1, 5.3, and 4.6; degrees of freedom = 82, 111, and 67, respectively, for each mouse). On average, the vessel diameter decreased by 6.6% in the sonicated region and increased by 10.3% in the contralateral region. Our analysis revealed that vasoconstriction was more prevalent in the treated region, whereas vasodilation was more predominant in the contralateral region. We did not find significant correlation between the extent of vessel diameter change and the initial diameter, as indicated by an R-squared value less than 0.15 (Figures S3C and S3D).

### Flow speed reduction following Mb-FUS in the open-skull study

**Figure 3:**
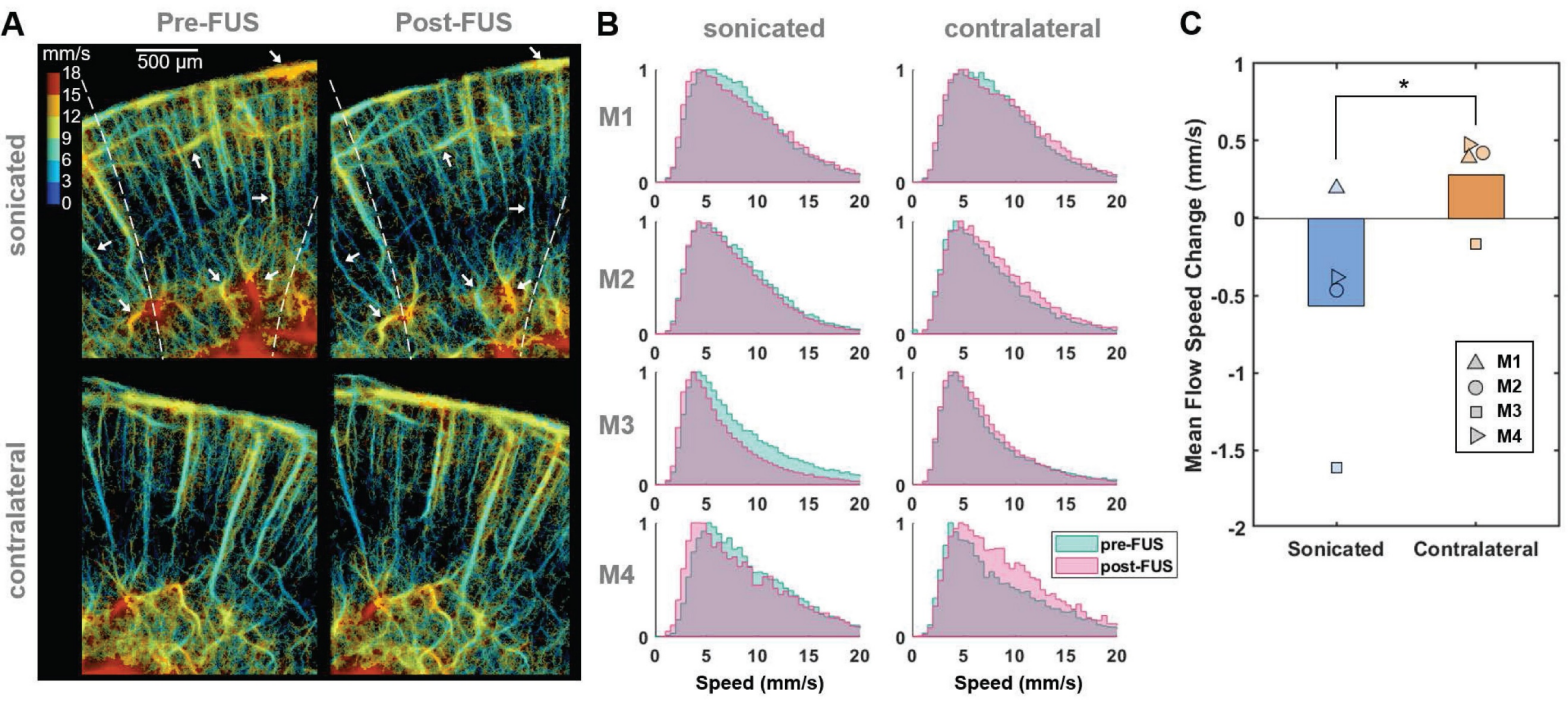
** Reduced microbubble flow speed in vessels at the sonicated side after FUS.** (**A**) Representative flow speed maps acquired from one of the craniotomized mice (M3) before (left) and after (right) Mb-FUS at the sonicated (top panels) and contralateral (bottom panels) regions. White arrows indicate vessels demonstrating a reduction in flow speed after FUS in the sonicated side. Dashed lines indicate the -12 dB FUS beam region. (**B**) Normalized histograms of flow speeds for tracked microbubbles in each mouse (M1-M4), comparing pre-FUS (green) and post-FUS (pink). The histograms exhibit a slight leftward shift (indicating a decrease in speed) after Mb-FUS in the sonicated region and a rightward shift in the contralateral region. (**C**) A bar graph for mean flow speed changes across four mice, showing a decrease in the sonicated region and an increase in the contralateral region. The paired t-test confirmed a significant difference between the sonicated and contralateral regions with p = 0.045. Histograms and the mean speed changes were obtained from cortex and hippocampal regions at the FUS axis or the contralateral side.

To evaluate changes in blood flow speed following FUS, we tracked microbubbles moving through the vessels across multiple frames and measured their flow speeds. Figure 3A displays representative flow speed maps acquired from the sonicated and contralateral brain regions in a craniotomized mouse both pre-FUS and post-FUS. Some individual vessels within the sonicated region (white arrows in Figure 3A) exhibited a reduction in flow of 1-4 mm/s, while changes in flow speed were less noticeable in the contralateral region. Figure 3B presents the histograms of pre-FUS and post-FUS flow speeds and the average changes in flow speed, in each mouse, respectively. The histograms revealed an overall decrease in microbubble flow speed after FUS in the sonicated region and an increase on the contralateral side. The mean flow speed in the sonicated region either decreased or, at least, increased less in all mice compared to that in the contralateral region (Figure 3C). The difference in the speed change between the sonicated and the contralateral regions was statistically significant (-0.57% vs. 0.28% on average, paired *t*-test, *t*-value = 3.32, degree of freedom = 3, *p* < 0.05).

### Transcranial BBBO using Mb-FUS

**Figure 4:**
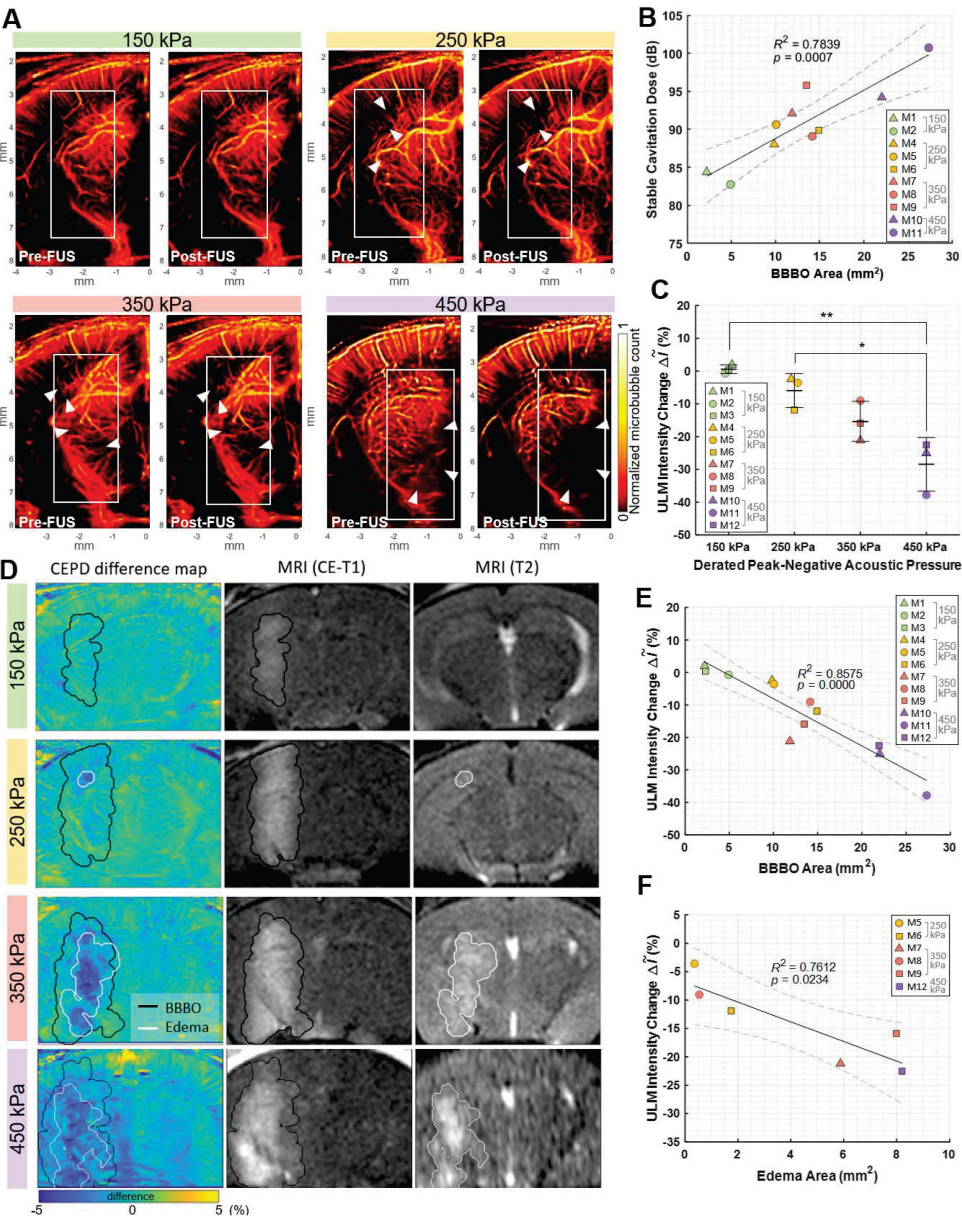
** Blood volume reduction after Mb-FUS in the transcranial experiments. A**) Representative pre-FUS (left) and post-FUS (right) ULM images for different acoustic FUS pressure groups (150, 250, 350, and 450 kPa). The colormap was power compressed for the better representation. **B**) Stable cavitation dose detected by PCD with respect to the BBBO area. **C**) Blood volume change detected from ULM images for different pressure groups (* *p* < 0.05, ** *p* < 0.01, one-way ANOVA). **D**) Representative CEPD difference maps, CE-T1 MRI (1 h after FUS), and T2 MRI (1 day after FUS) for pressure levels of 150, 250, and 350 kPa. BBBO region and edema region detected by CE-T1 and T2 MRI, respectively, are overlaid on the CEPD difference maps. **E**) ULM intensity reduction with respect to the BBBO area. **F**) ULM intensity reduction with respect to the edema area.

To investigate the transcranial feasibility of the method, CEPD and ULM images were acquired from the mouse brain with intact skin and skull before and after Mb-FUS. The left hippocampus and thalamus were sonicated at different acoustic pressures (150 kPa (*N*=3), 250 kPa (*N*=3), 350 kPa (*N*=3), and 450 kPa (*N*=3)) for BBBO by using a single spherical transducer, while the flow imaging was obtained by using the linear array transducer (Figure 1B). FUS parameters are listed in Table 1, while imaging parameters are listed in Table 2. BBBO was confirmed and quantified for all mice by CE-T1w MRI, and the different acoustic pressures resulted in various sizes of BBBO. The hyperintensity observed in T2-weighted (T2w) MRI was present in all three mice from the 350-kPa group, two out of three mice from the 250-kPa group, and was not detected in the 150-kPa group. As shown in Figure 4B, the size of BBBO was linearly correlated with the detected harmonic cavitation dose obtained from the passive cavitation detector (PCD) (Figure 1B). The sizes of BBBO and edema and the stable cavitation dose for each mouse are listed in Table S2. In all pressure groups, BBB was reinstated to baseline in 3-7 days confirmed by CE-T1w MRI.

### Transcranial detection of localized microbubble count reduction

Transcranial ULM images before and after sonication for each pressure group were presented in Figure 4A. The reduced image quality in transcranial ULM compared to open-skull imaging is attributed to well-known skull-induced effects, including acoustic attenuation and phase aberration 27,28. The ULM intensity change for each mouse is listed in Table S2. There was a greater reduction in the number of detected microbubbles in cases with higher pressure (white arrowheads). The average intensity (i.e., the normalized microbubble count) within the white box in Figure 4A was measured as the blood volume, revealing a greater reduction as pressure increased (Figure 4C). An ANOVA analysis showed a statistically significant difference among pressure groups (*F*-value = 14.42, df1 = 3, and df2 = 8). The reduction in blood volume measured by ULM showed a strong linear correlation with the size of the opening (*R*² = 0.86, *p* < 0.01) and a moderate correlation with the size of the edema (*R*² = 0.76, *p* < 0.03), as illustrated in Figures 4E and 4F, respectively.

CEPD difference maps after Mb-FUS were compared with CE-T1w and T2w MRIs for three pressure groups (Figure 4D). Quantified BBBO and edema regions from MRIs were overlaid on the difference map as black and white contours, respectively. The maps once again demonstrated a greater signal reduction in a broader area for higher FUS pressure. The localized region of blood volume reduction (blue in the map) roughly corresponded to the hyperintensity of T2w MRI for the 250 kPa, 350 kPa, and 450 kPa cases. However, the CEPD signal reduction within the BBBO contour was not consistently evident, with many pixels exhibiting values within the noise level. In the case of 150 kPa, where no T2 hyperintensity was found, there was no pronounced local reduction in the CEPD map. This result indicates that the sensitivity of the current transcranial CEPD may not be sufficient to detect BBBO without edema.

Similar to the observations in the craniotomy study, the reduction was particularly pronounced in regions where small vessels are distributed (Figure S4). We also confirmed that the larger differences in the small vessel regions are not attributed to division by a small number when computing percent changes (Figure S5). This observation may indicate that Mb-FUS has a greater impact on small vessels compared to larger ones, as we have found in immunohistochemistry and single-cell RNA sequencing 32.

From the histopathological evaluation of brain tissue using H&E staining FUS (Figure S6), no visible signs of hemorrhage or structural tissue damage were observed in the 150 kPa, 250 kPa, or 350 kPa groups. However, in the 450 kPa group, minor red blood cell extravasation was detected on the sonicated side. These results indicate that the CEPD signal reduction can be observed after FUS BBBO even in the absence of hemorrhage.

### Transcranial detection of flow speed reduction following Mb-FUS

**Figure 5:**
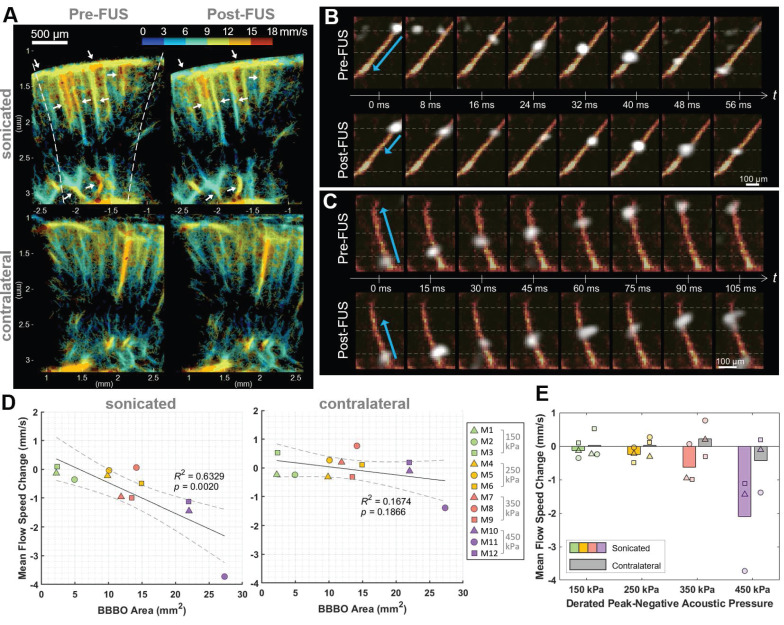
**Flow speed alteration after Mb-FUS measured by transcranial ultrasound flow imaging.** (**A**) Representative flow speed maps with the quantized colormap, transcranially obtained pre-FUS and post-FUS, showing more reduction in flow in the sonicated region (white arrows) compared to the contralateral region. Dashed lines indicate the -12 dB FUS beam region. (**B, C**) Timelapsed snapshots show microbubbles (white) flowing through vessels (orange-red) in (B) a mouse from the 250 kPa group and (C) a mouse from the 350 kPa group. The blue arrows indicate the distance traveled within the same timeframe. The horizontal gray dashed lines assist in gauging the traveled distance. Post-FUS microbubbles (second row of B and C) traveled more slowly compared to the pre-FUS ones (first row of B and C). Supplementary videos are available online as Movie S2 and Movie S3. (**D**) Mean flow speed change in the sonicated (left panel) and the contralateral (right panel) regions following Mb-FUS for all mice with respect to the size of BBBO. Linear regression lines and their 95% confidence intervals are presented as solid and dashed lines, respectively. (**E**) Group-wise analysis of the mean flow speed change across different acoustic pressure groups. The bar graphs indicate the average change within each group. The average flow speed showed a reduction in the sonicated region compared to the contralateral region, with the extent of reduction increasing with the pressure.

The flow speed reduction following Mb-FUS was also observed through the transcranial ultrasound flow imaging. Figure 5A shows the flow speed maps in the cortical and hippocampal regions of the sonicated and contralateral hemispheres before and after FUS. Slowed flow was observed (white arrows in Figure 5A) in more vessels on the sonicated than on the contralateral side. Figures 5B and 5C show the tracked movement of individual microbubbles at each time point through vessels in mice from the 250 kPa and 350 kPa groups, respectively, and supplementary videos are available (Movie S2 and Movie S3). They visually demonstrate a microbubble traveling through a vessel after FUS more slowly than another bubble passing the same vessel before FUS. The mean flow speed changes after FUS were evaluated within ROIs of the sonicated and contralateral regions. In most cases, a decrease in mean flow speed was noted in the sonicated region, with the reduction linearly correlated to the size of the BBBO (*R*² = 0.63, *p* < 0.01) (Figure 5D). In contrast, no significant trend was identified in the contralateral region (*p* > 0.1). When analyzed by pressure group (Figure 5E), the greater reduction in flow speed at the sonicated region was observed as the acoustic pressure of FUS increased.

## Discussion

The significance of this study lies in employing CEPD and ULM as innovative tools for assessing the effects of Mb-FUS on vascular dynamics. For the investigation of the cerebrovascular response to FUS, previous studies primarily relied on microscopy, MRI, and fMRI, providing insights at a limited depth or employing costly imaging modalities. To our knowledge, the application of ultrasound flow imaging has not been explored in the context of FUS-induced BBBO. This study demonstrated the promising potential of ultrasound imaging for assessing Mb-FUS effects on cerebrovascular dynamics, offering improved penetration depth, cost-effectiveness, and potential integration into ultrasound-guided FUS systems. To observe the immediate response to FUS, we used ultrasound flow imaging with microbubbles, which allowed us to capture post-FUS vascular changes without the need to wait for microbubble clearance. In this study, we established an ultrasound approach to monitor and quantify vascular changes following Mb-FUS in mice. We also demonstrated, for the first time, that transcranial ultrasound imaging can detect reductions in flow volume and speed, which are associated with the size of the opening and edema. In both open-skull and transcranial experiments, we observed decreases in both the number of detected microbubbles and their speed at the sonicated region after Mb-FUS. Furthermore, average vessel diameter measured by ULM through a cranial window decreased at the sonicated region after FUS.

While we utilized ULM and CEPD to measure blood volume, vessel diameter, and flow speed, it is important to acknowledge potential measurement error inherent to contrast-enhanced flow imaging. Unlike power Doppler (PD) imaging without microbubbles, which correlates with the quantity of moving red blood cells and indicates local blood volume 33, CEPD and ULM primarily reflect the distribution and dynamics of circulating microbubbles, rather than providing a direct measurement of true blood volume. Furthermore, microbubble characteristics, including size, concentration, perfusion, and stability, could introduce variability in the ULM signal intensity, vessel diameter, flow speed measurements 28,34. The variability inherent in microbubble localization over time in ULM also affects the reproducibility of vascular dynamics measurements.

To mitigate this variability, we used pre-FUS and post-FUS images with similar microbubble concentrations by selecting datasets with the same range of CEPD signal intensity (Figure 1C). Additionally, when comparing pre- and post-FUS, we normalized the averaged signal intensity in the sonicated region by that of the contralateral region. In vessel diameter measurements, we employed the averaging of cross-section profiles along a 50 μm length to address variability introduced by the stochastic distribution of microbubbles within the vessel.

Overall, our results show reductions in vessel diameter and flow speed following Mb-FUS, partially aligning with findings reported in other studies utilizing optical microscopy and MRI. Studies employing microscopy in mice 20 and rats 19 observed a prevalence of vasoconstriction over vasodilation as a response to Mb-FUS, which are consistent with our findings. In contrast, Burgess *et al.* reported more vessel dilation than constriction in mice. While Cho *et al.* observed greater constrictions in smaller vessels, our investigation did not reveal a strong relationship between the extent of diameter change and the vessel size (Figures S3C and S3D). These discrepancies may stem from differences in imaging depths (0-0.3 mm vs. 0-5 mm), FUS parameters and sequences, and craniotomy timepoints, warranting further investigation.

In the context of blood flow speed, a study using microscopy reported a delayed perfusion of Evan's Blue dye in a mouse after Mb-FUS (9 s vs. 4 min) 20. Stupar *et al.*'s study using pCASL MRI reported a substantial (~50%) reduction in cerebral blood flow lasting at least 1.5 h following FUS-induced BBBO with edema in rats 22. Additionally, Labriji *et al.* demonstrated a transient cerebral perfusion decrease in rats, reaching its lowest point at approximately -30% after FUS without causing edema 23. While our study also observed a reduction in flow speed (5-15%) at the sonicated hemisphere, it was not as pronounced as MRI studies. Particularly in the 150-kPa group, where no edema was detected, the reduction in flow speed was not detectable compared to the contralateral side.

This discrepancy may stem from several factors, such as differences in the studied species (e.g., variations in vasomotor responses between mice and rats; 35), time frames for imaging (5-10 min vs. 1-2 h), or differences in sensitivity and mechanisms between the two imaging modalities. Especially in our study, the mean flow speed measured by ULM would reflect larger vessels more than smaller ones due to the higher likelihood of detecting bubbles in larger vessels. Additionally, ULM is a motion-based technique, and the ranges of detectable velocities are biased, possibly leading to less accurate estimates in smaller vessels with slower speeds. This characteristic of ULM would have contributed to the low sensitivity, if the reduction primarily occurred in small vessels and capillaries.

None of the prior studies exploring vascular changes after Mb-FUS has shown a reduction in blood volume, whereas our study observed a localized blood volume reduction in the presence of edema. Labriji *et al.* reported no significant change in cerebral blood volume as detected by dynamic susceptibility contrast MRI, possibly due to the absence of edema cases in their investigation. In contrast, our analysis of transcranial CEPD images revealed a notable local reduction in blood volume near the edema site in 4 out of 5 mice exhibiting T2 hyperintensity, with a linear correlation between the blood volume reduction and the size of edema. The reduction in CEPD signal may indicate vessel disruption, vasospasm, and ischemia, potentially leading to vasogenic and cytotoxic edema with inflammatory responses 36-38. Given that such changes could impair local oxygen delivery and metabolic support to brain tissue, their hemodynamic consequences warrant further investigation.

Another interesting finding was that the reduction in the microbubble count was particularly observed in regions with small vessels (< 20 μm) (Figure S4). This phenomenon may be because the transient occlusion or vasospasm of upstream vessels could induce a further reduction or temporary cessation of blood flow in downstream vessels. Additionally, microbubble oscillation might have caused more extensive stretching of smaller vessels compared to larger ones 39, resulting in a greater impact on smaller vessels. While one study showed that BBB in larger capillaries (6-10 μm) was easier to disrupt than that of smaller capillaries 40, another study focusing on the larger scale of vessels (0-100 μm) revealed that majority of leaky vessels following FUS were smaller than 25 μm 41. Furthermore, Nhan *et al.* reported that fast leakage (i.e., high permeability rate) is more prevalent in small vessels (10-30 μm), potentially indicating a higher likelihood of microdisruption for smaller vessels under Mb-FUS 42. This may explain our observation of blood volume reduction and co-localized edema.

While the observation of reduced flow following FUS BBBO is consistent with prior findings, our study provides new findings enabled by the higher resolution of ULM, which is the detection of changes in vessel diameter. While the low resolution of CEPD (i.e., CEUS) could not resolve the small vessels that are mainly responsive to the FUS, the high resolution of ULM powered by the localization of microbubbles provided enough spatial resolution to measure the vessel diameter.

An opposite response in the contralateral hemisphere compared to the treated side was observed; vessel dilation and increased flow speed. While this phenomenon could be attributed to measurement variability due to the limited sample size, it may also reflect a compensatory or autoregulatory response to the stimulation. Further investigation is needed to determine the underlying mechanisms driving this effect.

We observed larger BBBO with edema at similar acoustic pressure levels used in our prior studies 3,43-45. This may stem from various factors, such as a different skull-attenuation assumption (18% vs. 20%), a longer pulse duration (0.67-6.7 ms vs. 10 ms), an extended sonication time (1 min vs. 2 min), and a higher microbubble dose resulting from residual bubbles from the initial injection for pre-FUS imaging, given that longer sonication and a higher microbubble dose have been associated with larger openings and stronger immune response 43,46. Despite the promising findings, our study has several limitations that warrant further investigation. The first limitation was the craniotomy on the same day as the experiment, which could have led to brain swelling and inflammation. The brain swelling after craniotomy affected spatial registration between pre- and post-FUS flow images, as well as between flow images and MR images. Although we initiated data acquisition 30 min after the craniotomy to allow the initial brain swelling to subside, a subtle but gradual swelling persisted. While the movement within 5 min during consecutive dataset acquisitions was negligible (< 6 μm), the displacement between pre- and post-FUS images with a time gap of ~20 min was 30-50 μm. Furthermore, the non-rigid deformation of the vascular structure due to the swelling made registration challenging. Additionally, variability in the targeting depth of FUS across mice may have contributed to further differences in the observed outcomes.

Second, minor tissue damage along the craniotomy margin during the procedure led to gadolinium leakage, which was detected on the cortical surface near the margin (Figure 2B). Consequently, our analysis focused on the hippocampal region, where BBB opening was directly attributed to FUS, excluding cortical areas affected by surgical artifacts. Also, the inflammation resulting from the craniotomy might have impacted vascular dynamics, contributing to the variability observed in the open-skull study results. Implanting an acoustic-permeable cranial window (i.e., chronic cranial window models) to enable post-surgery imaging would aid in mitigating these confounding factors in future studies. Nevertheless, the reduction in both blood volume and speed observed in the open-skull study was also replicated in the transcranial study with intact skin and skull.

Additionally, this study lacks the temporal observation of vascular dynamics after FUS over time. The microscope studies revealed the dynamic vessel caliber change such as a rapid constriction followed by recovery and sometimes dilation within 5-15 min 19-21, while MRI studies showed the spatiotemporal evolution of blood flow change over 1-1.5 h 22,23. Additionally, Labriji *et al.* observed a medial-to-lateral propagation of cerebral perfusion decrease along the cortex, indicating a potential association with cortical spreading depression (CSD). Given other recent intriguing findings on CSD following FUS 47,48, it seems valuable to explore the temporal evolution of vascular changes following FUS. However, in this study, the long data acquisition time (> 5 min) of ULM prevented the examination of transient changes in vessel diameter or flow speed. In future studies, spatiotemporal vascular dynamics will be explored by employing advanced ULM techniques such as dynamic ULM 49 or microbubble uncoupling/separation methods 50,51.

Another limitation of this study is the relatively small sample size per group, which may affect the generalizability of the findings. Although a statistically significant correlation between BBB opening or edema size and blood volume reduction was observed, larger sample sizes in future studies will be necessary to improve statistical power, detect more subtle effects, and reduce inter-subject variability.

Our findings warrant further exploration and consideration of potential applications in FUS therapy. First, ultrasound flow imaging using CEPD and ULM can serve as a complementary monitoring tool alongside cavitation-based techniques such as PCI and passive acoustic mapping (PAM). Cavitation monitoring provides real-time mapping of acoustic emissions and is widely used to estimate cavitation dose and spatial targeting during FUS procedures 13,26,52,53. However, it primarily captures the acoustic energy generated by oscillating microbubbles and does not directly reflect the resulting biological or vascular effects. In contrast, CEPD and ULM offer insights into microbubble-induced changes in blood volume, flow speed, and vessel diameter, which are more directly associated with biological outcomes. For example, in our study, regions showing signal reduction in ULM co-localized with edema observed on T2w MRI, whereas PCI showed higher acoustic energy in larger vessel regions. The two modalities provide distinct but synergistic information: PCI reflects cavitation behavior, which is critical for real-time sonication control, while flow imaging captures the downstream physiological impact of cavitation. By adding flow imaging capabilities, ultrasound-guided systems become more comprehensive and self-sufficient, accelerating the clinical translation of compact and cost-effective FUS treatments.

Lastly, recent achievements in transcranial ultrasound flow imaging in humans have demonstrated promising potential for clinical translation. Notably, the feasibility of acquiring ULM images through the human temporal bone has been demonstrated 54, and significant progress in aberration correction and motion correction algorithms 55,56 and SNR improvement technique 57 is expected to accelerate clinical translation.

## Conclusions

We hereby established a method to quantify changes in blood volume, flow speed, and the vessel diameter following Mb-FUS using ultrasound flow imaging with microbubbles in mice. Our findings indicate that Mb-FUS induces a reduction in blood volume and flow speed at the treated region, with vasoconstriction being more pronounced than vasodilation. Additionally, we demonstrated the transcranial capability of CEPD and ULM to detect the vascular changes after Mb-FUS by observing linear relationships between the flow signal reduction and the size of opening or edema. This is the first time that ultrasound can image the blood vessels that experience BBBO and visualize flow changes and potential damage, together with cavitation mapping. These findings not only provide novel insights into the vascular response to FUS-induced BBBO but also offer a cost-effective and clinically translatable approach for real-time monitoring of FUS interventions at the microvascular level.

## Materials and Methods

### Animals

The animal studies were conducted in compliance with the guidelines established by the Institutional Animal Care and Use Committee (IACUC) of Columbia University and were approved by the same committee. Wild-type male C57BL/6 mice aged 6-10 weeks (The Jackson Laboratory, Bar Harbor, ME, USA) were used in the study. For the open-skull study, a total of four mice (N = 4) were used, and craniotomy was performed from bregma +0 to bregma -4mm with a width of 8 mm under anesthesia with 2.0-2.5% isoflurane. The data acquisition for the mice was initiated at least 30 min after the completion of the craniotomy. For the transcranial study, twelve mice were used and divided into four groups, each exposed to different acoustic pressures: N = 3 (150 kPa), N = 3 (250 kPa), N = 3 (350 kPa), and N = 3 (450 kPa). Their heads were shaved and depilated while the scalp and skull remained intact. During imaging and FUS sonication, mice were anesthetized with 1.5-2.0% vaporized isoflurane mixed with oxygen (1 L/min) and the body temperature was regulated by using a heating pad at 36-38°C. A 27-gauge butterfly needle was inserted into the tail vein to facilitate intravenous (IV) injections of saline or microbubbles solutions for both imaging and BBBO.

### Experimental setup

We utilized two distinct experimental setups for open-skull and transcranial experiments. The open-skull study provided high-quality imaging for accurate vascular measurements, while the transcranial study evaluated the feasibility of FUS through the intact skull for future applications. In the open-skull study, we employed the same linear array transducer (L22-14vXLF; number of elements: 128, transmit frequency: 15.6 MHz) for both imaging and therapy using a theranostic ultrasound (ThUS) sequence 58. The mice, which were anesthetized and had undergone craniotomy, were secured in a stereotaxic frame and imaging and sonication were performed through the cranial window with degassed acoustic coupling gel (centrifuged at 2000 rpm for 20 min), as illustrated in Figure 1A. A research ultrasound system (Vantage 256; Verasonics Inc., Kirkland, WA, USA) was used for controlling the FUS transmit sequence and acquiring the ultrasound image data.

For the transcranial study, a single-element spherical FUS transducer (diameter: 60mm, focal depth: 60 mm, transmit frequency: 1.5 MHz) was employed for BBBO, and the 15.6-MHz linear array transducer was used for transcranial imaging (Figure 1B). Anesthetized mice had their heads secured and shaved. Degassed gel was applied over the scalp, and a degassed water bath was positioned above the mouse head to ensure acoustic coupling with the transducers. The spherical transducer and the linear array were aligned horizontally using a 3-D printed holder and connected to a 3D positioner. The array was initially placed on the mouse head for pre-FUS imaging and then replaced with the spherical FUS transducer for BBBO using the 3D positioner. Immediately after FUS, the array was returned to the same position for post-FUS imaging. The spherical FUS transducer was driven by a function generator (Keysight, Santa Rosa, CA, USA) through a power amplifier (325LA; E&I, Rochester, NY, USA) to generate therapeutic pulses, while the linear array was controlled by the research ultrasound system to acquire ultrasound images. In all experiments, the linear array was positioned at the center of the coronal brain slice at bregma -2 mm by the guidance of B-mode and Doppler imaging.

### Microbubbles

Polydisperse microbubbles were used for both BBBO and flow imaging. The microbubbles were synthesized in-house based on 1,2-distearoyl-sn-glycero-3-phosphocholine (DSPC, Avanti Polar Lipids Inc., Alabaster, AL, USA) and 1,2-distearoyl-sn-glycero-3-phosphoethanolamine-N-[methoxy(polyethylene glycol)-2000] (DSPE-mPEG2000, Avanti Polar Lipids Inc., Alabaster AL, USA), following previously published protocols 44,58,59. A vial of the lipid solution with perfluorobutane gas was activated by using a shaker (VialMixTM, Lantheus Medical Imaging, MA, USA) to form polydisperse microbubbles on the same day as the experiment. The in-house microbubbles herein have been characterized in previous studies, demonstrating their efficiency for BBB opening compared to commercial microbubbles 44,60. Their lipid composition including DSPG enhances membrane stability 61, ensuring greater durability for flow imaging. The mean diameter and the concentration of the microbubbles were 1.76 μm and 7.7×10^9^ microbubbles/ml. The microbubble solution was diluted to a concentration of 4×10^8^ microbubbles/mL before use. A 100-μL bolus of the solution was injected for pre-FUS imaging, followed by another 100-μL bolus for Mb-FUS around 10 min after the first injection. Additional microbubble solution was injected for post-FUS imaging, depending on the CEPD intensity.

### Focused ultrasound for BBBO

For BBBO with the imaging transducer in the open-skull study, we used the ThUS sequence as described in 58, utilizing electronically-focused ultrasound with a short pulse. Given that the transmit frequency of the probe we used here was 10 times higher than the frequency used in the previous study (15.6 MHz vs. 1.5 MHz), the focal size was only ~0.1 mm in width with an F-number of 1 (the number of transmit elements: 50). To compensate for the small focal size, we transmitted 5 foci spanning 0.5 mm in the lateral direction (blue arrow in Figure 1A). The sonication sequence and parameters are presented in Figure S1A and Table 1. The simulated acoustic beam patterns of the single focus and the 5 foci are shown in Figure S2. The number of bursts was 60, and the burst repetition frequency was 0.5 Hz (i.e., 2 min of total sonication time). In each burst, 100 pulses per focus were sonicated with a pulse repetition frequency of 1 kHz. The 5 pulses for the 5 foci were transmitted with a between-foci interval of 17 μs considering the round-trip time for the depth of 10 mm. The mechanical index (MI) of the focused beam was 0.6, and the peak negative pressure was 2.3 MPa. The left hippocampus and the upper (dorsal) part of the thalamus were targeted for BBBO, with the focus set at 2.5 mm deep from the cortical surface and 2 mm caudal from bregma.

For the conventional FUS sonication with a single-element transducer in the transcranial study, a 10-ms long pulse was transmitted for 2 min with a PRF of 2 Hz (Figure S1B, Table 1). The FUS frequency was 1.5 MHz, and the derated pressure of FUS ranged from 150 to 450 kPa, assuming skull-induced attenuation of 20%. The focus was placed at 3-4 mm deep from the cortical surface, 2-2.5 mm left from medial, and 2-2.5 mm caudal from bregma, covering the left hippocampus and thalamus.

In both open-skull and transcranial studies, a 100-μL bolus of microbubbles were intravenously administered for BBBO with a concentration of 4×10^8^ microbubbles/mL immediately after the start of the sonication. The peak negative pressure was verified through free-field acoustic measurements in water using a hydrophone (HGL-0200, Onda Corp., Sunnyvale, CA, USA).

### Acquisition and reconstruction of CEPD and ULM images

In both the open-skull and transcranial studies, we used the same imaging sequence to acquire CEPD and ULM images approximately 10 min before and after Mb-FUS (Figure 1C). Pre-FUS images were obtained after a 100-μL bolus injection of microbubbles. The low-resolution CEPD image (pixel size: 0.2mm×0.2mm) and the CEPD intensity averaged over a field-of-view (5 mm×9 mm) were displayed for real-time monitoring of the bubble concentration in the mouse brain. With another bolus injection, FUS was sonicated for 2 min to open the barrier at the left hippocampus and thalamus. After sonication, additional microbubbles were injected and post-FUS flow images were obtained.

For both CEPD and ULM, we utilized plane wave compounding with 9 steering angles to acquire a dataset consisting of 500 frames with an effective frame rate of 1 kHz (Table 2). Multiple datasets were obtained within 5-10 min before and after Mb-FUS. Datasets within a similar range of CEPD intensity (highlighted in yellow in Figure 1C) were chosen for reconstructing pre-FUS and post-FUS images, under the assumption that CEPD intensity is proportional to microbubble concentration. This assumption was made considering that signal intensity and imaging quality with microbubbles would be affected by their concentration in the brain. Approximately 80 consecutive datasets (~8 min) were selected and used for reconstructing a single frame of CEPD or ULM.

High-resolution CEPD with a pixel size of 50 µm × 50 µm and super-resolution ULM with a pixel size of 6.25 µm × 6.25 µm (~λ/16, where λ is the wavelength of the imaging ultrasound) images were reconstructed offline. Inphase-quadrature (IQ) beamforming was used to form the ultrasound image 62, and singular value decomposition (SVD) filtering with a cutoff of 20-30 (i.e., axial flow speed < 1-1.5 mm/s) was applied to the IQ-beamformed images to remove the tissue and breathing motion 28. A representative SVD-filtered ultrasound video of microbubble flow is provided as a supplementary video (Movie S4). We obtained CEPD images by squaring the pixel intensity of the filtered images and averaging all the frames of multiple datasets. In the case of ULM, the IQ beamformed images were reconstructed with a pixel size of 25 µm × 25 µm (~λ/4) and processed by SVD filtering. The microbubble separation was applied by using the positive and negative Doppler frequency bandpass filters 63. The filtered images were interpolated by a factor of 2 and deconvoluted using a Gaussian filter (standard deviation: 50 µm×50 µm). To localize microbubbles, the *imregionalmax* function in MATLAB (The MathWorks, Natick, MA) was employed 64,65, after thresholding at the 0.95 quantiles of pixel intensity and interpolating again by a factor of 2. The final ULM images with the pixel size of λ/16 were obtained by summing the number of detected microbubbles within an image pixel across multiple datasets. Microbubbles were paired between consecutive frames using the Hungarian algorithm, and only tracks longer than 10 frames were retained for flow speed measurement 66. To enhance robustness, microbubble pairing between alternative frames (i.e., the *k*-th and (*k*+2)-th frames) was also allowed. The high-resolution CEPD and ULM images were reconstructed offline, with the processing times for generating a compounded frame being approximately 30 min and 3 h, respectively. The vessel saturation curves for ULM image reconstruction were presented in Figure S7.

### Analysis of CEPD and ULM Images

ULM intensity (i.e., number of detected microbubbles within each pixel) was averaged within a ROI centered at the FUS focus. Then, the averaged intensity was normalized by that of the contralateral region; 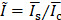, where 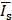
and 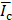
are the averaged intensities within ROIs at the sonicated and contralateral hemisphere, respectively. The percent change of the intensity following FUS was measured by 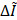
(%) = 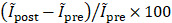, where 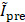
and 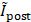
are the normalized averaged intensities in pre-FUS and post-FUS images, respectively. The change in ULM intensity after Mb-FUS was compared with the acoustic pressure and the sizes of BBBO and edema in the transcranial experiment analysis. Note that the rectangular ROI does not represent the exact size or shape of the focal region. Instead, the acoustic intensity profile of the FUS beam is presented in Figure S2.

Vessel diameter was measured for specific vessel segments selected in the sonicated and the contralateral regions under the criteria: each segment is well-reconstructed in both pre-FUS and post-FUS ULM images, not overlapping with other vessels, and is longer than 50 μm. For each segment, fifteen cross-section profiles perpendicular to the vessel direction were obtained along the length of 50 μm with an interval of 2 μm. The diameter of each segment was estimated by averaging the cross-section profiles and measuring its full-width half-maximum. One mouse (Mouse 1) was excluded from the vessel diameter measurements due to an insufficient number of ultrasound datasets with matched CEPD intensity.

For microbubble flow speed analysis, only the cortex and hippocampal regions were examined due to challenges in separating and tracking individual bubbles in the regions with a dense vasculature, such as the thalamus. The flow speed histogram and the mean flow speed change were obtained from microbubble tracks within a 2 mm (lateral) × 2.5 mm (axial) ROI, covering both the cortex and hippocampal regions and aligning with the axis of FUS focus, which is the field of view of Figure 5A.

### MRI

We acquired MRIs to confirm BBB opening and assess the edema (9.4T Ascend, Bruker Medical, Billerica, MA). For the detection and quantification of BBBO, CE-T1w MRI was obtained approximately 1 h after Mb-FUS and 30 min after the intraperitoneal injection of a gadolinium-based MR contrast agent (Omniscan, Princeton NJ; 0.2 mL per mouse). T2w images were also obtained 1 day after Mb-FUS without contrast enhancement for assessment of edema. The parameters of the scans are presented in Table S1.

In the open-skull study, the confirmation of BBBO in the cortical part was challenging due to inflammation resulting from the craniotomy. However, in the deeper region near the focus, spanning the hippocampal and upper thalamus regions, we confirmed the opening by identifying contrast-enhanced regions with intensities notably higher than those observed in the contralateral hemisphere.

For the comparison with the 2-D ultrasound flow images, a 2-D coronal slice of MRI corresponding to the B-mode and ULM images was reconstructed and used for the quantification. The BBBO region was quantified from CE-T1w MRI with a threshold of two standard deviations above the mean pixel intensity in the contralateral hemisphere, while the edema region was obtained from T2w MRI with a threshold of one standard deviation above the mean intensity. The thresholds used to detect BBBO and edema were determined to ensure that the visually identifiable hyperintensity regions were adequately captured. Pixels with intensities higher than a threshold were selected and the selected area was filtered using erosion and dilation filters to eliminate small false-positive areas 3.

### Cavitation monitoring

The PCI was obtained in the open-skull study, where the linear array transducer was used for both imaging and therapy (Figure 1A), as in the previous studies 14,31,58. A single PCI per burst was obtained using the following equation:


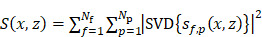

(1)

where 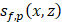
represents the delay-and-sum beamformed image for the *f*-th focus and the *p*-th pulse and SVD{·} denotes the SVD filtering. The 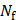
and 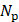
are the number of foci and pulses, respectively, and in this study, they were 5 and 100. In SVD filtering, the beamformed data for each focus *f* were rearranged into a 2D space-time Casorati matrix 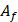
of size (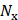
×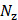, 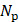), where 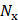
×
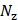
is the number of imaging pixels. The first 10 singular values were discarded to remove stationary reflections and slow-moving tissue and flow 67, and the last 10 singular values were also excluded to reduce noise. The beamformed data 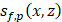
were derived as follows:


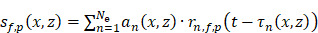

(2)

where 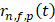
is the RF data received by the *n*-th transducer element for the *f*-th focus and the *p*-th pulse, 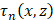
is the round-trip delay, 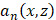
is the apodization coefficient with a Hamming window, and 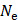
is the number of elements. The round trip delay was determined as the sum of transmit delay, 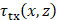, and the receive delay, 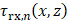, where 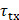
is the time delay of the focused ultrasound wave to arrive at the imaging point and 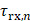
is the time delay from the imaging point to the *n*-th element. The 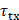
was obtained by applying a Gaussian filter with a standard deviation of 0.5 mm to the arrival time map generated using the '*computeTXPD*' function in the Verasonics system. Real-time PCI per burst was displayed during FUS sonication, and the cumulative PCI map was generated by integrating the PCI maps across all bursts.

In the transcranial study, the cavitation dose was monitored by using the PCD shown in Figure 1B. The stable cavitation dose was measured from the 3^rd^ to 7^th^ harmonic frequencies. The stable cavitation dose was calculated by summing the squared peak amplitudes of the 3^rd^ to 7^th^ harmonic frequencies and taking the square root of the sum 26.

### Statistical analysis

Statistical analysis was conducted using MATLAB (The Mathworks Inc., Natick, MA) or GraphPad Prism (GraphPad Software Inc., La Jolla, CA). For the open-skull study, diameter changes in vessel segments at the sonicated and contralateral regions were compared using an unpaired t-test due to the non- matching vessel segments between regions. Mean flow speed changes in the sonicated and contralateral regions were compared using a paired t-test. For the transcranial study, linear regression analysis was employed to investigate the relationships between stable cavitation and BBBO, mean ULM intensity change and BBBO size, mean ULM intensity change and edema size, as well as mean flow speed change and BBBO size. R-squared values and p-values were computed to assess the goodness-of-fit and statistical significance of the model using. One-way ANOVA was used to assess ULM intensity changes among the four pressure groups.

## Supplementary Material

Supplementary figures and tables, movie legends.

Supplementary movie 1.

Supplementary movie 2.

Supplementary movie 3.

Supplementary movie 4.

## Figures and Tables

**Table 1:** Parameters for FUS sonication for BBBO in the open-skull and transcranial experiments

	Open-skull experiment	Transcranial experiment
**Transducer**	Linear array probe (L22-14vX-LF)	Single-element, spherical transducer
**Frequency**	15.6 MHz	1.5 MHz
**Focal depth**	5 mm	60 mm
**F#**	1	1
**Pressure**	2.3 MPa	150-450 kPa (derated)
**Num. of foci**	5	1
**Num. of cycles**	5	15,000 (10 ms)
**Num. of pulses**	100 per focus	240
**Num. of bursts**	60	1
**Assumed skull-induced attenuation**	N/A	20%

**Table 2:** Parameters for ultrasound flow imaging (CEPD and ULM) in both open-skull and transcranial experiments

Imaging parameters	
**Num. of PWs**	9
**PW angle interval**	1°
**Sampling rate**	62.5 MHz
**Ensemble length (i.e., num. of frames per dataset)**	500
**Effective framerate**	1 kHz
**SVD filter cutoff**	30

## Abbreviations

BBB: Blood-brain barrier
BBBO: Blood-brain barrier opening
CEPD: Contrast-enhanced power Doppler
CSD: Cortical spreading depression
FUS: Focused ultrasound
IACUC: Institutional Animal Care and Use Committee
IQ: Inphase-quadrature
IV: Intravenous
Mb-FUS: Microbubble-mediated focused ultrasound
MI: Mechanical index
MRI: Magnetic resonance imaging
pCASL: Pseudo-continuous arterial spin labeling
PD: Power Doppler
PCI: Power cavitation imaging
ROI: Region-of-interest
SVD: Singular value decomposition
ThUS: Theranostic ultrasound
ULM: Ultrasound localization microscopy
