# Supplementary material for: Ultrasound flow imaging for assessing cerebrovascular changes following focused-ultrasound blood-brain barrier opening
Source: Theranostics. 2025 Sep 22;15(18):10028–44. doi: 10.7150/thno.98098 (PMC12486155; doi:10.7150/thno.98098)
Supplement: Supplementary file 1 — Supplementary figures and tables, movie legends. [file thnov15p10028s1.pdf]

Supplementary Materials for

Ultrasound Flow Imaging for Assessing Cerebrovascular Changes Following Focused-Ultrasound Blood-Brain Barrier Opening

Sua Bae, Stephen A. Lee, Seongyeon Kim, Fotios Tsitsos, Yangpei Liu, and Elisa E. Konofagou

Included in this PDF File:

Figures S1 to S7
Tables S1 and S2
Captions for Movies S1 to S4

Additional Supplementary Materials:

Movies S1 to S4

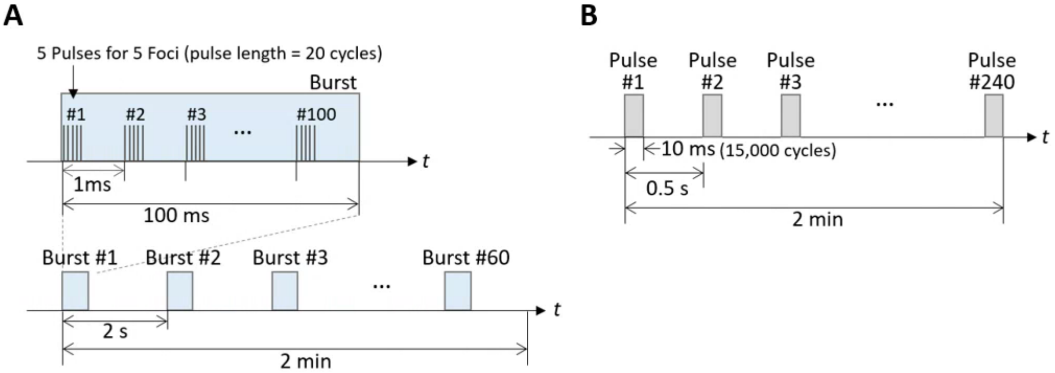

Figure S1. **Focused ultrasound (FUS) pulse sequences for blood-brain barrier opening (BBBO)**. (**A**) Short-pulse FUS sequence for the open-skull experiment using a linear array transducer with a center frequency of 15.6 MHz. Five focal spots with a lateral interval of 0.5 mm were sequentially sonicated to induce the larger opening than the size of focus. Sixty bursts were transmitted for 2 min with a burst repetition frequency (BRF) of 0.5 Hz, and each burst comprises of 100 pulses per focal spot with a pulse repetition frequency (PRF) of 1 kHz. (**B**) Conventional long-pulse FUS sequence for the transcranial experiments using a single-element spherical transducer with a center frequency of 1.5 MHz. A 10-ms-long pulse was transmitted for 2 min with a PRF of 2 Hz.

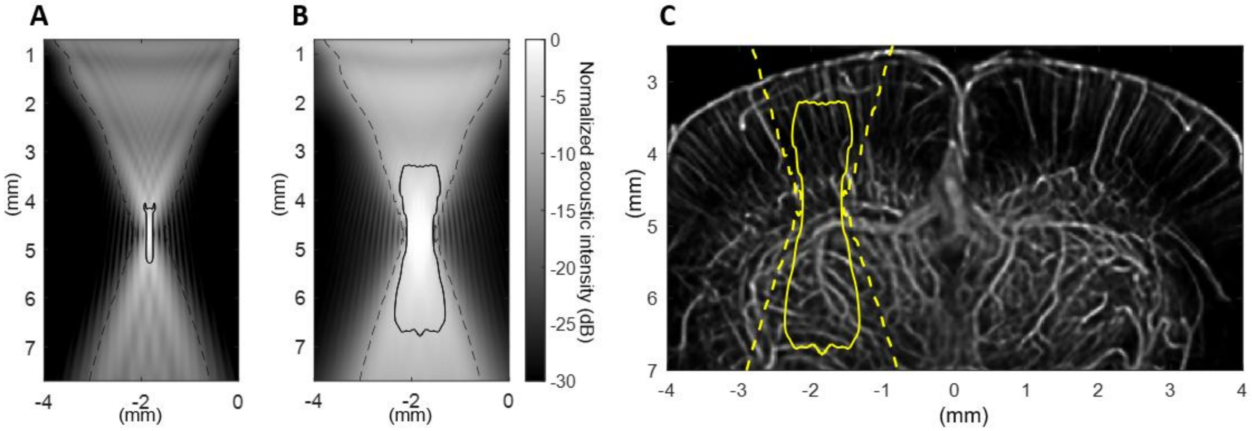

Figure S2. Simulated FUS beam patterns used for BBB opening in the open-skull study with the imaging probe. (A) Simulated acoustic beam pattern from a single transmit event. The solid black line indicates the −6 dB beamwidth contour. (B) Simulated compounded beam pattern using five transmit foci, which were used for BBB opening in the open-skull study. The −6 dB and −12 dB beamwidth contours are shown as solid and dashed black lines, respectively. (C) The −6 dB and −12 dB beamwidth contours from (B), overlaid on the ULM image, to illustrate the beam coverage relative to the vascular structures.

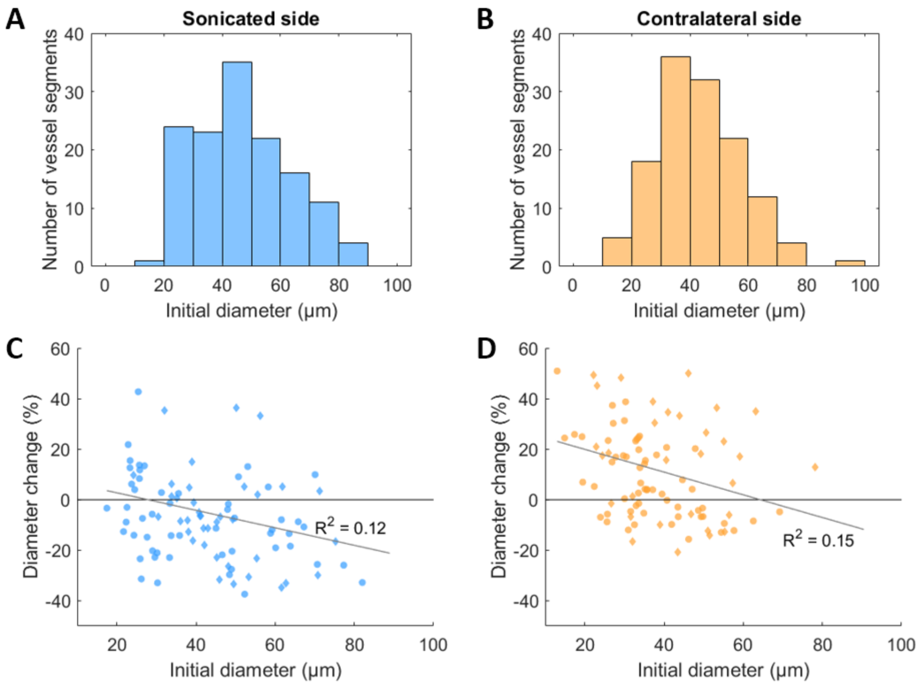

Figure S3. **Histogram of the initial diameters of vessel segments used for measuring diameter changes, and the relationship between the initial vessel diameter and the diameter change following FUS.** (**A, B**) Distribution of intraluminal diameters of vessel segments selected for diameter measurement in the sonicated (A) and contralateral (B) regions. (**C, D**) Relationship between the initial vessel diameter and diameter change following Mb-FUS in the sonicated (C) and contralateral (D) regions. The vessel segments from three mice were pooled into the plots. The segments were selected considering only those that were well-reconstructed in both pre-FUS and post-FUS images. Due to reduced flow after FUS, particularly pronounced in small vessels, some small vessels on the sonicated side were not well-reconstructed post-FUS. This led to a larger mean diameter for vessels selected in the sonicated region compared to the contralateral region. The extent of vessel diameter change showed no correlation with the initial diameter, as indicated by an R-squared value less than 0.15.

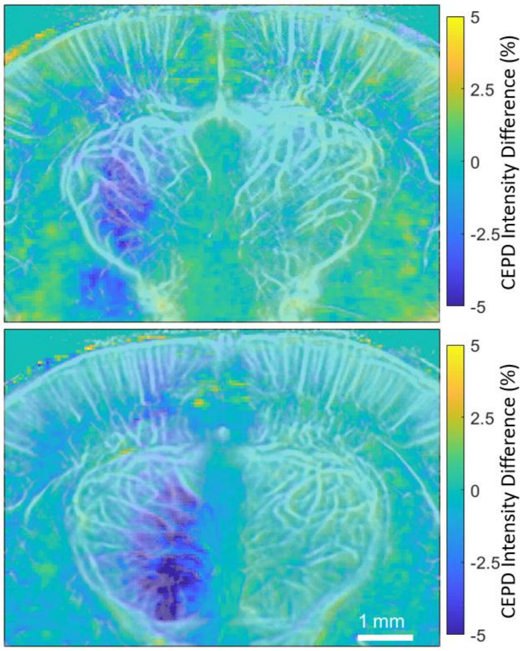

Figure S4. Vessel distribution (semi-transparent white) overlaid on the CEPD difference map (blue-yellow colormap). The reduction in CEPD intensity, indicated by blue, was prominent in areas where small vessels are prevalent.

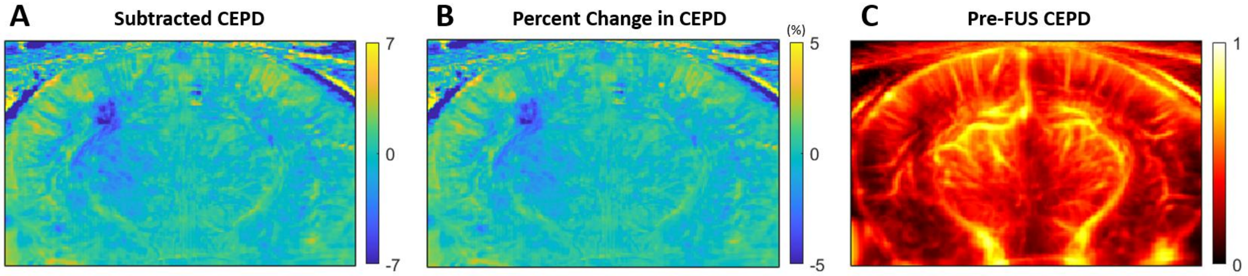

Figure S5. **Comparison between the subtraction map and the percent change map**. (**A**) Subtracted CEPD map ($I_{\text{post}} - I_{\text{pre}}$), (**B**) CEPD percent difference map ($(I_{\text{post}} - I_{\text{pre}})/I_{\text{pre}} \times 100$), and (C) pre-FUS CEPD map showing the small and large vessel regions. I_{pre} and I_{post} are each pixel value of pre-FUS and post-FUS CEPD maps, respectively. As the subtracted map (A) was similar to the percent difference map (B), we confirmed that the greater signal reduction at the smaller vessel region in the CEPD percent difference map was not solely due to the small denominators (i.e., lower signal intensities in smaller vessels) when computing percent changes.

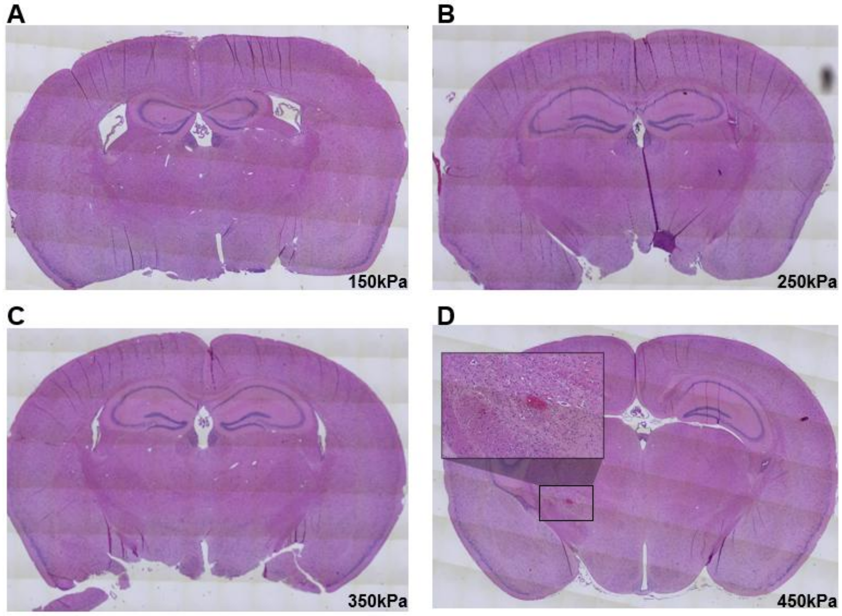

Figure S6. Histological evaluation of brain tissue using H&E staining following FUS at pressure levels of (A) 150 kPa, (B) 250 kPa, (C) 350 kPa, and (D) 450 kPa. No signs of hemorrhage or tissue damage were observed in the 150 kPa, 250 kPa, or 350 kPa groups. However, minor red blood cell (RBC) extravasation, indicative of slight hemorrhage, was observed on the sonicated side in the 450 kPa group. The inset in (D) provides a magnified view of the region.

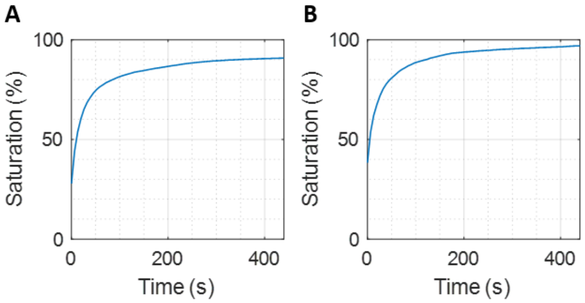

Figure S7. Vascular saturation curves for ULM images obtained from (a) the open-skull study and (b) the transcranial study, illustrating the completeness of microvascular detection over an 8-minute period.

Table S1. Parameters for contrast-enhanced T1-weighted MRI and T2-weighted MRI

Sequence Name	Contrast-enhanced T1-weighted (CE-T1w)	T2-weighted (T2w)
Repetition time (ms)	230	2500
Echo time (ms)	3.3	10
Number of averages	6	6
Flip angle (°)	70	
In-plane resolution (mm)	0.1×0.1	0.1×0.1
Slice thickness (mm)	0.4	0.57

Table S2. Transcranial experiment results with different acoustic pressures. ULM intensity change (i.e., change in the number of detected microbubbles) was measured from ULM images, while BBBO and edema sizes were measured from the T1-weighted and T2-weighted MRIs, respectively.

Group	Mouse#	BBBO size (mm^2)	Stable cavitation dose (dB)	ULM intensity change (%)	Edema size (mm^2)
150	1	2.2	84.4	1.95	0
	2	4.9	82.8	-0.70	0
	3	2.3	*	0.37	0
250	4	9.9	88.0	-2.47	0
	5	10.1	90.6	-3.58	0.36
	6	14.9	89.9	-11.89	11.74
350	7	11.9	92.1	-21.19	5.87
	8	14.2	89.1	-9.05	0.53
	9	13.5	95.8	-15.89	7.99
450	10	22.0	94.2	-25.07	*
	11	27.3	100.7	-37.80	*
	12	21.9	*	-22.57	8.20

*Data were not acquired.

Movie S1. Power cavitation imaging (color) during FUS sonication overlaid on the vessel map (grayscale) obtained through a cranial window.

Movie S2. Microbubbles (white) flowing down through a vessel (orange-red) in a mouse from the 250 kPa group. The microbubble captured post-FUS (right) traveled slower than the one captured pre-FUS (left). Horizontal gray dashed lines assist in gauging the traveled distance in the same time frame.

Movie S3. Microbubbles (white) flowing up through a vessel (orange-red) in a mouse from the 350 kPa group. The microbubble captured post-FUS (right) traveled slower than the one captured pre-FUS (left). Horizontal gray dashed lines assist in gauging the traveled distance in the same time frame.

Movie S4. A representative SVD-filtered ultrasound video of microbubble flow.
